# MiR-132 inhibition improves myocardial strain in a large animal model of chronic left ventricular adverse remodelling

**DOI:** 10.1093/ehjimp/qyaf088

**Published:** 2025-08-04

**Authors:** Sandor Batkai, Andreas Spannbauer, Janika Viereck, Celina Genschel, Steffen Rump, Denise Traxler, Martin Riesenhuber, Dominika Lukovic, Katrin Zlabinger, Ena Hasimbegovic, Thomas Thum, Mariann Gyöngyösi

**Affiliations:** Research and Development, Cardior Pharmaceuticals GmbH, Hollerithallee 20, Hannover 30419, Germany; Division of Cardiology, Medical University of Vienna, Waehringer Guertel 18-20, Vienna 1090, Austria; Research and Development, Cardior Pharmaceuticals GmbH, Hollerithallee 20, Hannover 30419, Germany; Research and Development, Cardior Pharmaceuticals GmbH, Hollerithallee 20, Hannover 30419, Germany; Research and Development, Cardior Pharmaceuticals GmbH, Hollerithallee 20, Hannover 30419, Germany; Division of Cardiology, Medical University of Vienna, Waehringer Guertel 18-20, Vienna 1090, Austria; Division of Cardiology, Medical University of Vienna, Waehringer Guertel 18-20, Vienna 1090, Austria; Division of Cardiology, Medical University of Vienna, Waehringer Guertel 18-20, Vienna 1090, Austria; Division of Cardiology, Medical University of Vienna, Waehringer Guertel 18-20, Vienna 1090, Austria; Division of Cardiology, Medical University of Vienna, Waehringer Guertel 18-20, Vienna 1090, Austria; Research and Development, Cardior Pharmaceuticals GmbH, Hollerithallee 20, Hannover 30419, Germany; Institute of Molecular and Translational Therapeutic Strategies (IMTTS), Hannover Medical School, Carl-Neuberg-Str. 1, Hannover 30625, Germany; Division of Cardiology, Medical University of Vienna, Waehringer Guertel 18-20, Vienna 1090, Austria

**Keywords:** heart failure, cardiac hypertrophy, strain imaging, cardiac MRI, adverse cardiac remodelling, microRNA-132

## Abstract

**Aims:**

Cardiac miR-132 has been proposed as a target for heart failure (HF) therapy. CDR132L, a rationally designed synthetic oligonucleotide inhibitor of miR-132 has proved pre-clinical efficacy in non-ischaemic and ischaemic large animal HF models. The safety and tolerability of CDR132L were tested in chronic HF patients in a Phase 1b study (NCT04045405) and is currently being tested in a Phase 2 trial in post-MI HF patients (NCT05350969). The aim of the current study was to gain further data on myocardial function and efficacy of CDR132L by analysing left ventricular (LV) and atrial (LA) wall motion by serial cardiac magnetic resonance (cMRI) strain imaging in a clinically relevant large animal (pig) model of chronic HF.

**Methods and results:**

Animals (15 per group) were randomized 1-month post-MI and received five intravenous (i.v.) monthly treatments with CDR132L (5 mg/kg) or placebo and were followed up for 6-month post-MI. LV and LA strain parameters were deteriorated after MI over time but significantly ameliorated by CDR132L treatment, compared with placebo. Strain parameters showed significant correlations with pharmacodynamic measures such as ejection fraction, NT-proBNP, and cardiac interstitial fibrosis in remodelling hearts 6 months post-MI.

**Conclusion:**

LV and LA motion and contractility were improved by repeated monthly dosing of CDR132L in a large animal model of HF with reduced ejection fraction model with first dose given one month post-MI. The results highlight the translational value and usability of MRI-based cardiac strain imaging in HF drug development and support further clinical development of CDR132L.

## Graphical Abstract

**Graphical Abstract:**
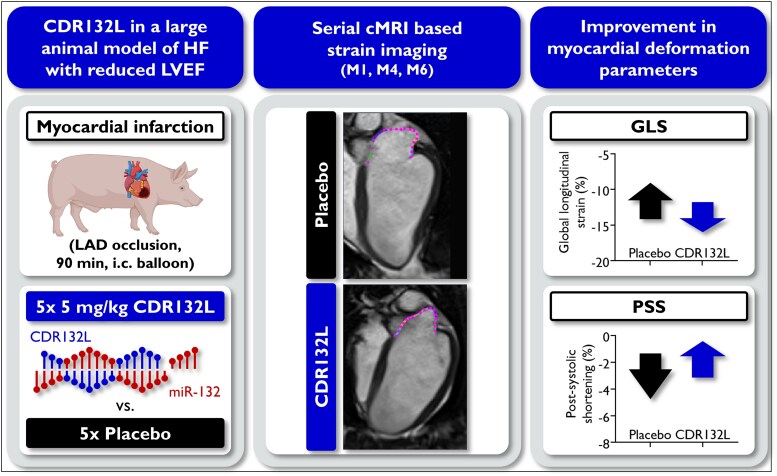
In a clinically relevant pig model of chronic post-MI heart failure, cardiac magnetic resonance (cMRI)-based strain imaging demonstrated that monthly CDR132L treatment, an oligonucleotide inhibitor of miR-132, significantly improved myocardial motion, reduced fibrosis, and correlated with enhanced cardiac function and stress biomarker changes. These findings support the therapeutic potential of CDR132L and highlight the translational value of strain imaging in heart failure drug development. Graphical element created in BioRender. Viereck, J. (2025) https://BioRender.com/8rfjou3.

## Introduction

With high mortality and re-hospitalization rates, the global burden of ischaemic-driven chronic heart failure (HF) is steadily increasing despite improvements in the standard of care and represents a great unmet need. Despite the progress made to develop large animal models to bridge mode-of-action-based pre-clinical concepts with therapeutic efficacy^1^, a major obstacle is the scarcity of meaningful translational surrogate endpoints. Cardiac magnetic resonance imaging (cMRI) has become not only the gold standard clinical tool but also indispensable for studying disease mechanisms, development of potential drugs for HF in the pre-clinical setting. Adoption of the cMRI-based novel imaging biomarkers for therapeutic efficacy is, however, impeded by limited experience.

CDR132L is a rationally designed synthetic oligonucleotide miR-132-3p (miR-132) inhibitor currently in Phase 2 clinical development (HF-Revert, NCT05350969) to evaluate safety and efficacy in HF patients with reduced ejection fraction (HFrEF), early after myocardial infarction (post-MI). CDR132L has shown pre-clinical efficacy in various HF models.^2–4^ In a Phase 1b study in chronic HF patients, CDR132L was found safe and well-tolerated, providing sustained and dose-dependent decrease of the target miR-132 in plasma.^5^

In this study, we explored the cMRI-based feature tracking technology to assess mode-of-action-based efficacy of CDR132L. Cardiac strain parameters were evaluated by analysing cMRI data derived from a large animal study in chronic post-MI HF.^3^ The primary aim of this study was to compare the strain imaging parameters between the placebo vs. CDR132L cohort.

## Methods

### Large animal model of post-MI HF and treatment regimen

The study was carried out in Mangalica pigs, a Hungarian breed of domestic pigs. This pig is suited for chronic studies as other frequently used (e.g. Danish Landrace or similar) due to slower body weight growth rate. All procedures involving large animals have been reviewed and approved by the Animal Welfare Committee of the University of Kaposvar, Hungary and animal handling complied with the Hungarian Law on Animal Experimentation and the European Directive on the Protection of Animals used for Scientific Purposes (2010/63/EU). The pig model of chronic post-MI HF and study design has been previously described.^3^

**Figure 1:**
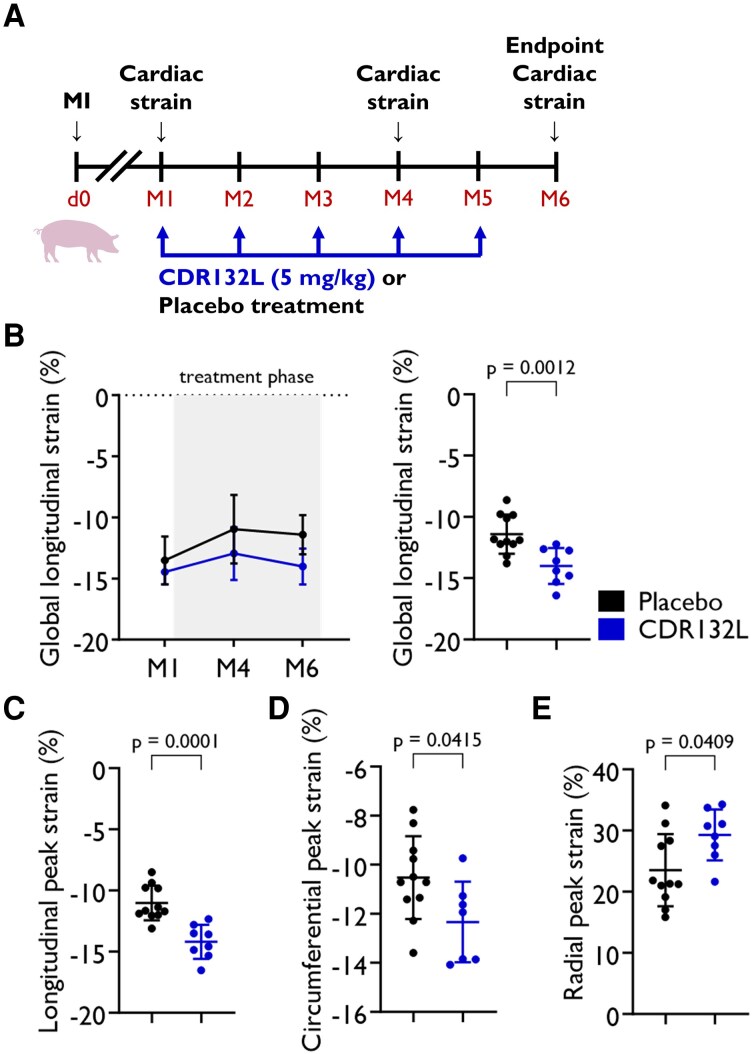
Study treatment regimens (*A*). Global longitudinal strain (*B*) over time and at endpoint (6 months). Longitudinal (*C*), circumferential (*D*), and radial peak strains (*E*) at endpoint (6 months). Data are mean ± SD; *P* values were calculated by two-tailed Mann–Whitney *U* test (placebo vs. CDR132L at 6-month endpoint). Graphical element adapted from SG-design/stock.adobe.com.

Briefly, MI was induced by coronary artery occlusion of the left anterior descending coronary artery by inflation with a balloon catheter for 90 min, followed by reperfusion via balloon deflation. Cardiac function was evaluated by cMRI (Siemens Magnetom Vision 1.5 Tesla field strength equipment) at indicated timepoints (*Figure 1A*). Based on body weights, initially 30 animals were randomly assigned into the two main treatment groups in a blinded fashion. Animals showing a left ventricular ejection fraction (LVEF) of >40% at Month 1 were excluded from the study. The rest received the first treatment one month post-MI by intravenous injection of 5 mg/kg CDR132L or placebo (*Figure 1A*, placebo *n* = 11, CDR132L *n* = 8). Treatment was repeated monthly. In total, five treatments were given.

### Analysis of cardiac strain by cMRI feature tracking

cMRI images were analysed by experienced observers blinded to the randomization using the Segment for Windows software (version 3.1; Medviso AB, Sweden^6^). Volumetric measurements [e.g. LVEF and left ventricular end-systolic volume (LVESV)] were performed, on short axis, two- and four-chamber view cine MRI images.

Left atrial size was measured using the biplane area length technique in two- and four-chamber views.^7^

ECG was simultaneously recorded during cardiovascular magnetic resonance (CMR). The transmission of the ECG sign was carried out with an active electrode supplemented with an amplifier. The sensors of the electrode were located to form a regular triangle. One electrode was fixed at the axis of the sternum-body between the fifth and sixth ribs on the right, the other one electrode was fixed between the third and the sixth ribs in the direction of the left elbow, and the third was placed on the sternum-head on right ahead. The animals were fixed in supine position along the exam. Pressure, volume, and surface/intracardiac ECG signals are recorded (LabChart software, AD Instruments, Colorado Springs, CO) and analysed with the PV-analysis software module.

Strain analysis was done in cine CMR images using the strain analysis module in Segment.^8^ The clinical reproducibility of feature tracking in cine MRI has previously been validated.^9^ A segmentation scheme for the left ventricle recommended by AHA^10^ was adapted in this study to calculate segmental strain. Briefly, in the short axis, three slices (basal, mid, and apical) were chosen, and endo-/epicardium was delineated at end-diastole. After adjusting LV rotation, the software calculated peak mean circumferential and peak mean radial strains for each segment and the means for global value. In the long axis, cine images in two-, three-, and four-chamber views were chosen at end-diastole. After delineation of endo- and epicardium, the software calculates peak longitudinal strain and peak mean radial strain for each segment and the means for global value. Left atrial strain was delineated in the four-chamber view at end-diastole, and strain parameters were measured using the RV strain function model in Segment, as recommended by the strain analysis manual.^8^

Early systolic lengthening (ESL) and post-systolic shortening (PSS) were measured in selected long axis view segments with fully automated software module. ESL was defined as any measurable positive systolic strain during early systole. If no systolic lengthening occurred, the ESL was zero. For the purpose of this study, the presence of ESL was tallied in each segment of each individual animal and expressed as rate of occurrence within each group. PSS was defined as the difference between peak negative strain in systole and peak negative strain in the cardiac cycle. PSS and PSS index (PSI) were calculated segmentally and averaged for each animal.

The PSI was calculated as: ([peak negative strain in cardiac cycle − peak negative strain in systole])/(peak negative strain in cardiac cycle) × 100.^11,12^

#### Fibrosis assessment

As reported previously,^3^ cardiac tissue samples from the left ventricular remote region were fixed in 4% formalin, embedded in paraffin, and cut into slices of 4–5 µm. For quantification, the slices were stained with Picrosirius Red. Microscopy photographs were recorded on an Olympus microscope IX83. Fibrosis was quantified based on collagen quantity by computerized planimetry using ImageJ (version 1.51). The threshold was determined from four stained sections per animal, and the mean value of identical samples of the same animal was calculated.

### Plasma miR-132 measurement

As described,^3^ blood was collected into EDTA tubes for plasma sampling and spun at 2500 rpm for 10 min. RNA was prepared according to the manufacturer’s instructions of the miRNeasy Mini Serum/Plasma Kit (Qiagen) and converted into cDNA using the TaqMan MicroRNA Reverse Transcription (RT) Kit (Thermo Fisher Scientific). Plasma miR-132 levels at Month 6 were assessed by quantitative real-time PCR (qRT–PCR) on a ViiA 7 Real-Time PCRSystem (Thermo Fisher Scientific) using the ABsolute Blue QPCR Mix (Thermo Fisher Scientific) and TaqMan MicroRNA Assays (Thermo Fisher Scientific; assay ID 000457 for miR-132-3p and assay ID 000200 for the spike-in cel-miR-39). Measurements were performed in technical duplicates and reported as mean. Plasma miR-132 levels were calculated applying the 2^−ΔΔCq^ method (2^−(Cq miR-132 – Cq cel-miR-39)^) and normalized to placebo levels at Month 6.

### Statistical analysis

Data are shown as mean ± SD. Therefore, *P* values were calculated using unpaired two-sided Mann–Whitney *U* test at endpoint (Month 6) or the χ^2^ test was used as indicated. Linear regression analysis was performed based on non-parametric Spearman correlation for the whole data set. Descriptive statistics and data analysis were done by using GraphPad Prism software (GraphPad Software, CA, USA).

## Results

In the chronic model of post-MI HF (*Figure 1A*), the ischaemia reperfusion injury led to impaired cardiac function, evidenced by strong reduction in LVEF.^3^ During the following 5-month treatment period, CDR132L significantly ameliorated the reduced global cardiac function, compared with placebo. Over time, absolute LVEF increased by 7.1% in the CDR132L group, compared with a 3.4% decrease in the placebo group, as reported previously.^3^

In our current study, we assessed the cardiac deformation parameters by MRI-based strain imaging over time after MI and tested the effects of CDR132L treatment. In line with the global left ventricular function parameters, our analysis revealed that global longitudinal strain (GLS) significantly worsened between randomization and 6-month endpoint in the placebo-treated group (−13.5 ± 1.9 vs. −11.4 ± 1.6; *P* = 0.0052; *Figure 1B*, *Table 1*). GLS was comparable to placebo at the beginning of treatment but remained at the same level over time in the CDR132L treatment group (−14.5 ± 1.0 vs. −14.0 ± 1.5; *Figure 1B*, *Table 1*). The difference between the groups was statistically significant (2.6% placebo vs. CDR132L; *P* = 0.0012; *Figure 1B*, *Table 1*). The therapeutic effect was detectable at all dimensions of peak strain signal (absolute value of longitudinal, circumferential, and radial peak strains; *Figure 1C–E*, Supplementary data online, *Figure S1*, and *Table 1*).

**Table 1:** Serial cMRI measurement of strain imaging parameter in a pig model post-MI chronic HF at indicated timepoints

	Placebo			CDR132L		
	Month 1	Month 4	Month 6	Month 1	Month 4	Month 6
Global longitudinal strain (GLS, %)	−13.5 ± 1.9	−11.0 ± 2.8	−11.4 ± 1.6	−14.5 ± 1.0	−12.9 ± 2.2	−14.0 ± 1.5**
Longitudinal peak strain (%)	−13.6 ± 1.6	−10.9 ± 2.9	−11.0 ± 1.4	−14.4 ± 1.0	−12.9 ± 2.2	−14.2 ± 1.4***
Circumferential peak strain (%)	−12.9 ± 4.1	−9.9 ± 2.5	−10.5 ± 1.7	−11.4 ± 2.4	−10.4 ± 1.7	−12.3 ± 1.6*
Radial peak strain (%)	32.2 ± 7.8	21.2 ± 7.3	23.5 ± 5.9	32.7 ± 3.6	27.8 ± 6.1	29.3 ± 4.2*
Post-systolic shortening (PSS, %)	−1.4 ± 0.6	−1.8 ± 0.6	−2.6 ± 1.1	−1.2 ± 0.5	−2.0 ± 0.9	−1.5 ± 0.8*
Post-systolic index (PSI, %)	10.6 ± 5.1	17.4 ± 6.6	23.1 ± 7.7	8.3 ± 3.3	15.4 ± 8.0	10.8 ± 5.7***
Peak atrial strain (PAS, %)	38.8 ± 8.1	36.7 ± 4.4	27.9 ± 5.3	38.6 ± 9.7	38.7 ± 10.3	34.8 ± 6.6*

Data are mean ± SD.

^*^ *P* < 0.05, **P < 0.01, ***P < 0.001; non-parametric two-sided Mann–Whitney *U* test (placebo vs. CDR132L at 6-month endpoint).

**Figure 2:**
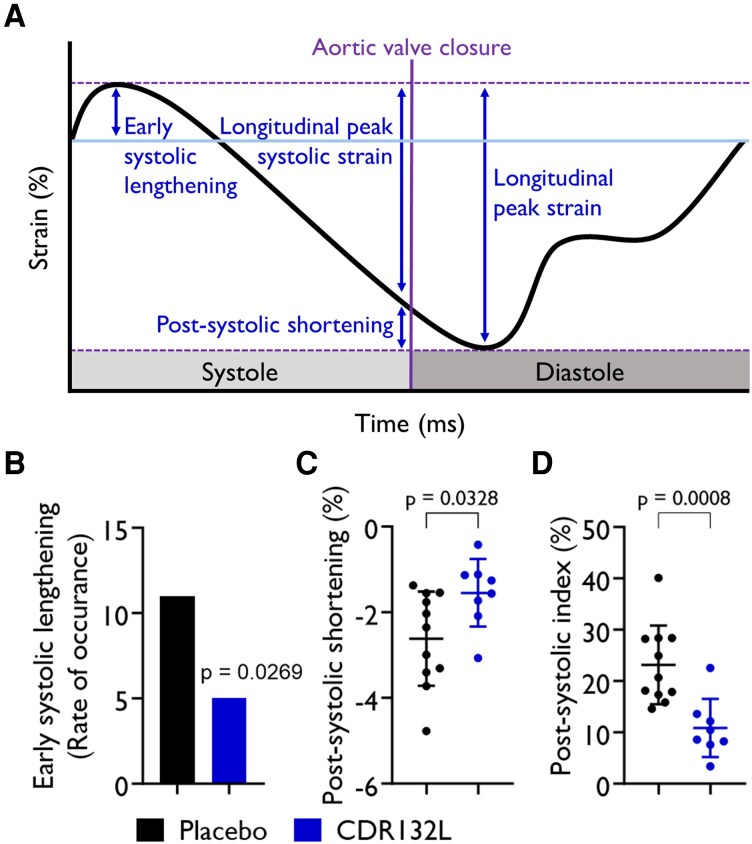
Schematic systolic ventricular strain signal with corresponding parameters that are relevant for systolic deformation (*A*). Frequency of early systolic lengthening (*B*), amplitude of post-systolic shortening (PSS) (*C*), and PSS index (*D*) at endpoint (6 months). Data are mean ± SD; *P* values were calculated by χ^2^ test (*B*) or two-tailed Mann–Whitney *U* test (*C*) (placebo vs. CDR132L at 6-month endpoint).

We also analysed patterns of anomalous myocardial deformation (*Figure 2A*), notably the ESL and the PSS (*Figure 2B–D*, Supplementary data online, *Figure S1*, and *Table 1*) in the remodelling myocardium. We identified the myocardial stretch during early systole (i.e. ESL) in both groups. The rate of occurrence was significantly higher in the placebo group vs. CDR132L-treated animals (*Figure 2B*, Supplementary data online, *Figure S1A*–*C*, and *Table 1*) at the 6-month endpoint.

PSS was also present in the remodelling hearts. We found that both PSS amplitude (−2.6 ± 1.1 vs. −1.5 ± 0.8; *P* = 0.0328; *Figure 2C*, Supplementary data online, *Figure S1*, and *Table 1*) and PSI (23.1 ± 7.7 vs. 10.8 ± 5.7; *P* = 0.0008; Supplementary data online, *Figure S1D* and *E*, and *Table 1*) were significantly different between CDR132L and the placebo groups.

**Figure 3:**
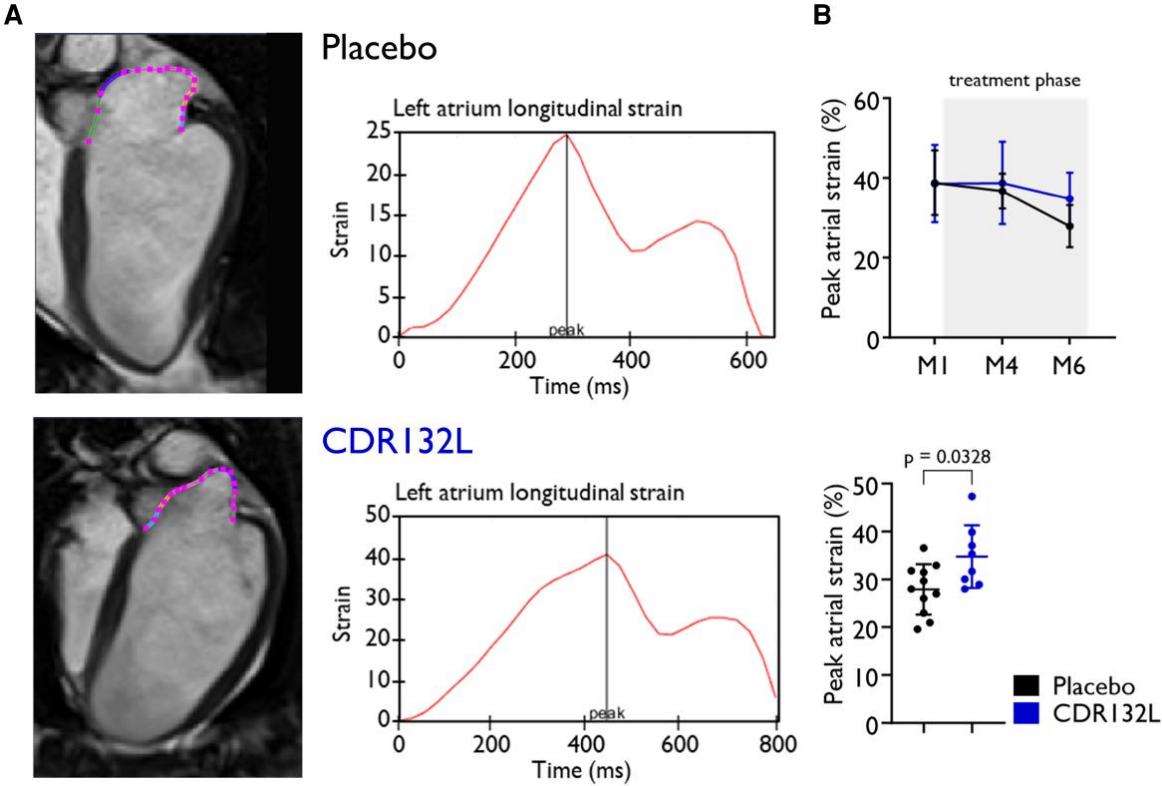
Representative images and recordings of longitudinal strain signal of the left atrium and peak atrial strain (*A*). Peak atrial strain (*B*) over time and at endpoint (6 months). Data are mean ± SD; *P* values were calculated by two-tailed Mann–Whitney *U* test (placebo vs. CDR132L at 6-month endpoint).

We also found evidence for pathological atrial remodelling in the hearts following MI (*Figure 3A*).^3^ In the CDR132L-treated group, left atrial volume (LAV) was significantly smaller in the CDR132L-treated group compared with the placebo-treated animals (LAV: 47.1 ± 10.2 vs. 34.8 ± 3.9, *P* < 0.01, respectively) at the endpoint, as reported previously.^3^ Regarding the wall deformation in the remodelled atrium, the strain analyses showed that the amplitude of peak longitudinal atrial strain (PAS) (*Figure 3A*) was also significantly increased in the CDR132L-treated animals (27.9 ± 5.3 vs. 34.8 ± 6.6; *P* = 0.032; *Figure 3B*, Supplementary data online, *Figure S1*, and *Table 1*) suggesting beneficial effects on atrial remodelling.

**Figure 4:**
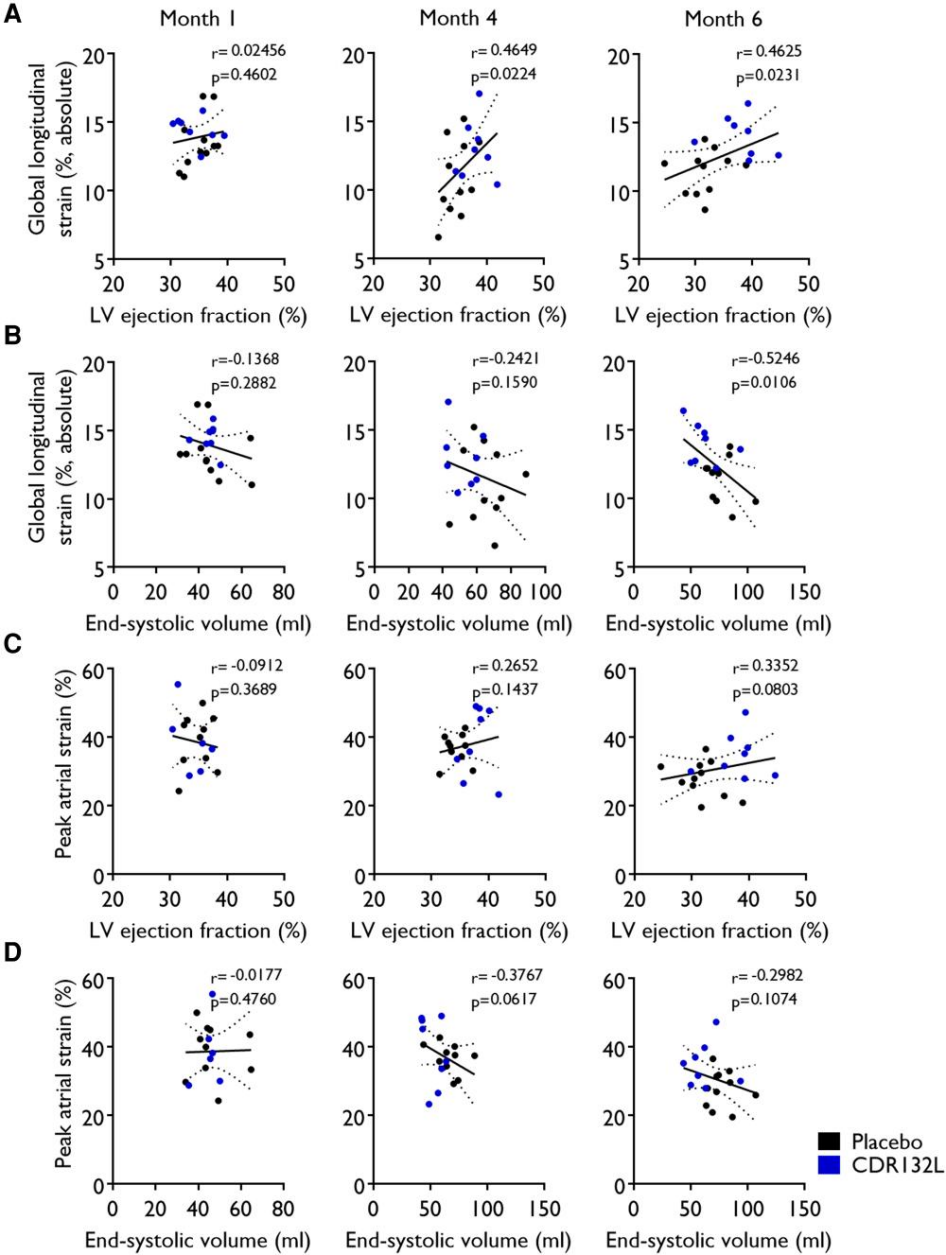
Correlation between global longitudinal (*A*, *B*) and peak atrial strains (*C*, *D*) vs. LV ejection fraction and LV end-systolic volume at different study timepoints. Correlation analysis was performed using non-parametric Spearman test (*r* and *P*).

Next, we analysed the relationship between pharmacodynamic parameters from our previous data set^3^ and the absolute value of LV and LA strain parameters across all animals. In general, the correlation between LVEF and LVESV with PAS and the absolute value of GLS was positive, whereas all other correlations were inverse (*Figure 4*, Supplementary data online, *Table S1*).

The absolute value of GLS had a significant positive linear correlation with LVEF at Month 4 (*r* = 0.4649; *P* = 0.0224) and Month 6 (*r* = 0.4625; *P* = 0.0231) and with LVESV at Month 6 (*r* = −0.5246; *P* = 0.0106), while the association of PAS was overall less strong, but a clear trend was recognized (*Figure 4A–D*, Supplementary data online, *Table S1*).

**Figure 5:**
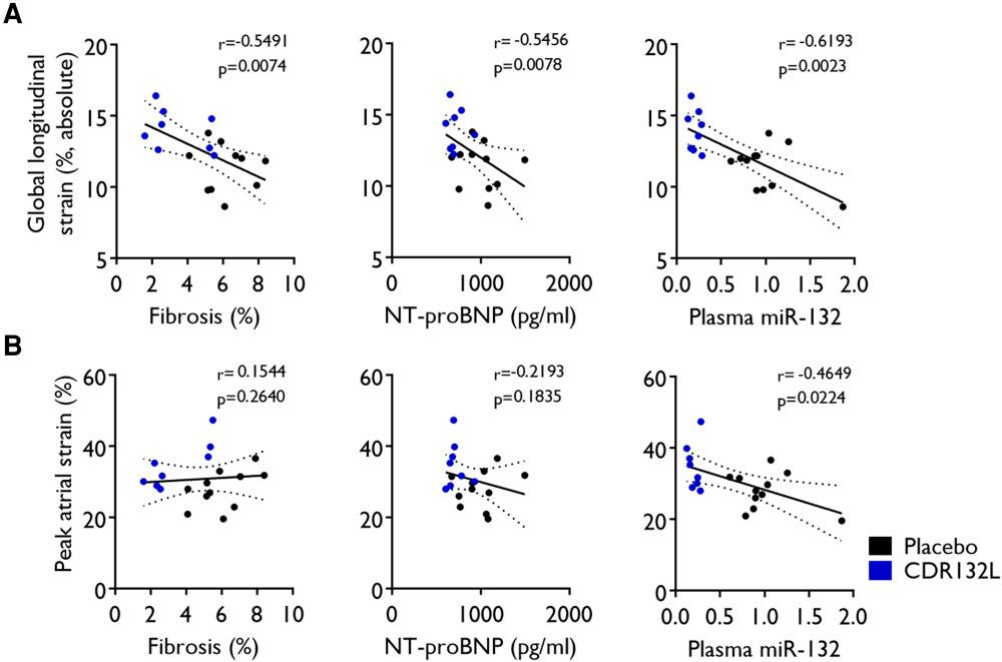
Correlation between cardiac fibrosis, NT-proBNP, or plasma miR-132 levels (2^−(Cq miR-132–3p – Cq cel-miR-39)^) normalized to placebo and (*A*) global longitudinal or (*B*) peak atrial strain at study endpoint (6 months). Correlation analysis was performed using non-parametric Spearman test (*r* and *P*).

Endpoint levels of markers of cardiac remodelling including the degree of interstitial cardiac fibrosis (*r* = −0.5491; *P* = 0.0074), and the stress biomarker NT-proBNP (*r* = −0.5456; *P* = 0.0078), had significant inverse linear correlations with GLS (*Figure 5A* and *B*, Supplementary data online, *Table S2*), while the correlation between atrial strain and such pharmacodynamic parameters showed a clear trend (*Figure 5*, Supplementary data online, *Table S2*). In addition, the magnitude of target engagement of CDR132L, measured as plasma miR-132 levels at endpoint, significantly correlated with the absolute value of GLS (*r* = −0.6193; *P* = 0.0023), and PAS (*r* = −0.4649; *P* = 0.0224) (*Figure 5*, Supplementary data online, *Table S2*).

## Discussion

Cardiovascular MRI-based strain imaging is a novel tissue tracking technique developed for non-invasive assessment of myocardial motion and deformation. This study aimed to evaluate mode-of-action-based pharmacodynamic effects of CDR132L using this imaging technique in a chronic model of adverse remodelling and post-MI HF in pigs. Cardiac strain parameters were evaluated by analysing cMRI data from a large animal study in chronic post-MI HF.^3^ To date, no similar data have been published in a model of chronic post-MI HF both in terms of size and study length.

GLS is an established imaging biomarker that strongly and independently predicts patient outcome.^13^ MRI-based GLS analysis exhibits superior image quality with less interference from anatomical structures^14^ and offers a more sensitive strategy for identifying dysfunction earlier in the natural history of HF.^15^ In a translational setting, high sensitivity and reproducibility were demonstrated in large animals.^16^

In our model, GLS worsened over time, paralleling the adverse cardiac remodelling in post-MI HF. The degree of change was in the same range with other published data in post-MI HF pig models.^17,18^ CDR132L-treated animals demonstrated better preservation of systolic performance, not only by improved LVEF^3^ but also by improvement of myocardial deformation, compared with the controls at the 6-month endpoint. This was clearly indicated by significantly improved GLS, and longitudinal, circumferential, and radial peak strains vs. control animals.

A similar pattern could be observed in a comparable large animal model.^18^ In that study, LVEF increase was accompanied by higher absolute GLS values after treatment with a sodium/glucose cotransporter 2 (empagliflozin).

We also analysed patterns of irregular myocardial deformation, notably the ESL and the PSS. We identified ESL, a characteristic paradoxical stretch during early systole in the remodelled hearts. PSS, a delayed myocardial contraction pattern that occurs after end-systole, was also detected in the post-MI hearts. CDR132L treatment significantly reduced the rate of ESL occurrence and PSS, as a sign of improved contractile function. ESL and PSS are an early and sensitive marker of myocardial dysfunction, in various forms of myocardial pathologies.^19^

We also evaluated CDR132L’s effect on atrial deformations in the remodelling hearts and found that LA strain was preserved in the CDR132L-treated animals. However, limitations of LA strain assessment in pigs, given their complex atrial anatomy and imaging challenges, need to be considered. The method applied in this study allowed accurate visualization of the LA, as previously published,^3^ while the data presented do not fully reflect LA size. Therefore, they were intended for wall tracking in strain analysis to highlight both its potential and the methodological challenges in large-animal imaging studies.

The absolute value of GLS demonstrated a significant correlation with several relevant pharmacodynamic endpoints. Correlation with interstitial fibrosis and NT-proBNP was moderate, with LVEF and LVESV somewhat less strong. The association between atrial strain and pharmacodynamic endpoints was less strong. We found the strongest correlation between PAS and LVEF, while with fibrosis and NT-proBNP, the relation was modest.

The superiority of myocardial deformation parameters over conventional parameters in predicting adverse cardiovascular events after MI has been indicated in clinical studies. MRI-based GLS is an independent predictor of all-cause mortality in HFrEF patients^20,21^ and strongly and independently predicted the occurrence of medium-term MACE in revascularized STEMI patients.^13^  ^,22^ The ability of PSS and ESL to predict adverse cardiovascular events in a spectrum of populations has also been recently shown.^19^ The incremental prognostic value over established cardiac risk factors after acute myocardial infarction has also been shown for left atrial strain imaging.^23^

We also took a closer look at the pharmacokinetic-pharmacodynamic relationship of miR-132 in this chronic model of HF. Systemic CDR132L application blocks its target, the miR-132 in in the heart, inducing therapeutic effect. Circulating miR-132 levels at endpoint provides a measure of target engagement. Indeed, we have previously demonstrated that circulating miR-132 levels highly correlate with cardiac miR-132, which in turn strongly correlated with the cardiac anti-remodelling effect in CDR132L-treated animals.^3,5^ In this study, we found that circulating miR-132 levels at the time of endpoint strongly correlated with both ventricular and atrial motion, measured as the GLS and peak atrial strain, respectively.

### Limitations and strengths

This is the first large animal study with longitudinal cardiac strain imaging, modelling chronic HF. Due to the use of a well-powered pig model of post-MI HF, state-of-the-art cardiac MR imaging and robust analysis, the data can be considered high quality and translationally relevant. However, the findings need to be considered in light of various limitations. As a general limitation, this study was performed on young animals that does not mirror clinical conditions for HF patients of older age. Another factor is sex differences in disease biology. Notably, the use of young female animals in this study must be taken into account for the interpretation of post-MI HF severity, evolution of LV adverse remodelling.

## Conclusion

In summary, this study is the first to provide data on myocardial motion and deformation in chronic endpoint (6 months) in post-MI remodelling hearts using MRI-based strain imaging in pigs. In support of our previous findings, we also provide pre-clinical evidence of the therapeutic effect of CDR132L, an inhibitor of miR-132, on LV mechanics. LV strain parameters were ameliorated in the treatment group and shoved significant correlation with cardiac function parameter, stress biomarker NT-proBNP, and with the reduction of cardiac interstitial fibrosis. Improvement of both ventricular and atrial measures of motion correlated well with the magnitude of target engagement by CDR132L application.

These mechanistic data indicate a pivotal role of miR-132 inhibition for the improvement of systolic-diastolic dysfunction of the failing heart. A treatment regime with as low as monthly frequency could be the basis for further clinical development of CDR132L in the broad area of chronic HF. Our results highlight the translational value and usability of MRI-based cardiac strain imaging biomarkers in heart failure drug development.

## Supplementary Material

qyaf088_Supplementary_Data

## Data Availability

The data underlying this article will be shared on reasonable request to the corresponding author.

## Lead author biography

Dr Sandor Batkai is a physician-scientist specializing in translational cardiovascular research, pharmacology, and pre-clinical drug development, with a particular focus on oligonucleotide and RNA therapeutics. In addition, he is an expert in cardiovascular phenotyping in cardiometabolic drug research including use of small and large cardiovascular disease animal models. His work bridges fundamental research and clinical application, aiming to advance novel therapeutic strategies for cardiovascular disease.
