# Supplementary material for: MiR-132 inhibition improves myocardial strain in a large animal model of chronic left ventricular adverse remodelling
Source: Eur Heart J Imaging Methods Pract. 2025 Aug 4;3(2):qyaf088. doi: 10.1093/ehjimp/qyaf088 (PMC12318716; doi:10.1093/ehjimp/qyaf088)
Supplement: qyaf088_Supplementary_Data [file qyaf088_supplementary_data.docx]

**Supplementary Data**

**MiR-132 inhibition improves myocardial strain in a large animal model of chronic left ventricular adverse remodelling**

Sandor Batkai MD, PhD^1,#,*^, Andreas Spannbauer^2,#^, Janika Viereck PhD^1#^, Celina Genschel PhD ^1^, Steffen Rump PhD ^1^, Denise Traxler^2^, Martin Riesenhuber^2^, Dominika Lukovic^2^, Katrin Zlabinger^2^, Ena Hasimbegovic^2^, Thomas Thum MD, PhD^1,3,*,#^, Mariann Gyöngyösi, MD^2,#^

**Supplementary Tables**

**Table 1:** Correlation analysis of global longitudinal and peak atrial strain with cMRI parameters at different timepoints.

|  |  | **Left ventricular ejection fraction (%)** | | | **End-systolic volume (ml)** | | |
| --- | --- | --- | --- | --- | --- | --- | --- |
|  |  | **M1** | **M4** | **M6** | **M1** | **M4** | **M6** |
| **Global longitudinal strain (%, absolute)** | r | 0.0247 | 0.4649 | 0.4625 | -0.1368 | -0.2421 | -0.5246 |
|  | p | 0.4602 | 0.0224 | 0.0231 | 0.2882 | 0.1590 | 0.0106 |
| **Peak atrial strain ( %)** | r | -0.0912 | 0.2652 | 0.3352 | -0.0177 | -0.3767 | -0.2982 |
|  | p | 0.3689 | 0.1437 | 0.0803 | 0.4760 | 0.0617 | 0.1074 |

r and p; nonparametric Spearman test.

**Table 2:** Correlation analysis of global longitudinal and peak atrial strain with other pharmacodynamic parameters at endpoint (M6).

| **Month 6** |  | **Fibrosis**  **(%)** | **NT-proBNP**  **(pg/ml)** | **Plasma miR-132**  **(relative to Placebo)** |
| --- | --- | --- | --- | --- |
| **Global longitudinal strain (%, absolute)** | r | -0.5491 | -0.5456 | -0.6193 |
|  | p | 0.0074 | 0.0078 | 0.0023 |
| **Peak atrial strain (PAS, %)** | r | 0.1544 | -0.2193 | -0.4649 |
|  | p | 0.2640 | 0.1835 | 0.0224 |

r and p; nonparametric Spearman test.

**Supplementary Figure**


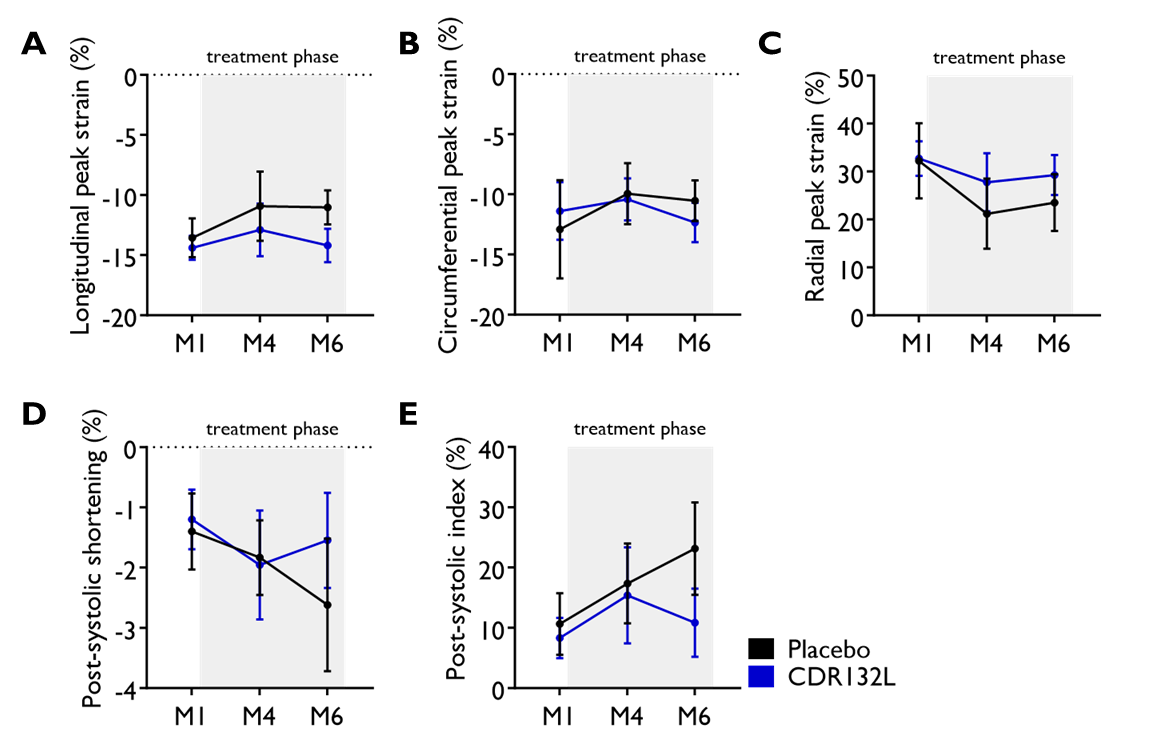


**Figure 1:** Strain imaging parameters over time. Data are mean ± SD.
